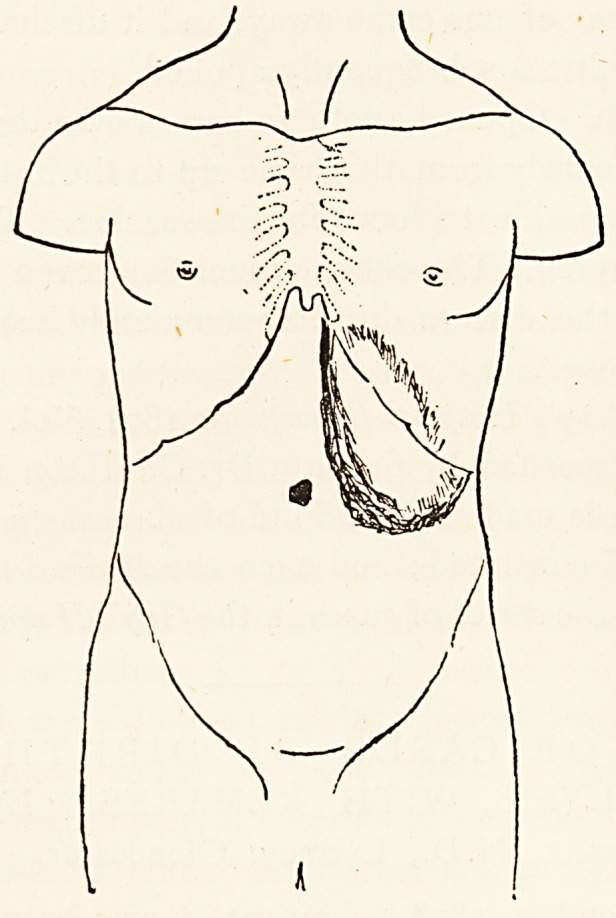# Case of Subdiaphragmatic Abscess of the Left Side

**Published:** 1884-09

**Authors:** J. Harper

**Affiliations:** Surgeon to the North Devon Infirmary, Barnstaple


### CASE OF SUBDIAPHRAGMATIC ABSCESS OF THE LEFT SIDE.

By J. Harper, L.R.C.P. Lond., Surgeon to the North Devon Infirmary, Barnstaple.

Abscesses in the upper part of the left side of the abdomen are of rare occurrence; they are interesting and anxious cases, and not many of them are recorded; this must be my excuse for troubling you with an isolated case, but it is the only one I have met with in my practice of over twenty-three years.

A male, 34 years of age, in the early part of August, 1876, contracted typhoid fever; it was a mild attack, and ran the usual course. He was convalescent and down stairs for about a week, when, from an error in his diet (over-feeding), he had a relapse, which commenced on October the 1st, and again ran through the regular course of the disease. During this second attack he had pleurisy of the left side, from which he speedily recovered, and was quite free from any chest affection. After being about for five weeks, he was suddenly seized with violent pain over the costal cartilages of the 7th and 8th left ribs. After a day or two a swelling the size of two fingers could be detected, extending from the ensiform cartilage towards the left side, behind and below the ribs. He was ordered to rest; the swelling covered with Ext. Belladonnæ; and after a day or two it seemed to get smaller. This was in the second week of February, 1877. After a day or two, without any apparent cause, the swelling commenced to increase in size, occupying nearly the whole of the left hypochondriac region, and extending downwards to an inch below the umbilicus, as shewn in the accompanying diagram.

On March 4th the swelling was beginning to extend upwards, over the cartilage of the ribs, and fluctuation was distinct. Dr. Richard Budd saw him with me, and we decided to use the aspirator. Nearly half a pint of thick, dirty-looking pus was drawn off, and the wound of the trocar covered with lint and collodion. The next day he was much the same; on the 6th the abscess was fuller; on March 8th the abscess was discharging freely through the trocar wound; pus looking healthy.

11th. The abscess was discharging; but as the matter was thick and occasionally plugging up the wound, I increased the size of the opening.

21st. The wound had again healed; the swelling was tense and very tender. I made a free incision, and about twelve ounces of pus came away, and it discharged freely into the poultices subsequently applied.

April 6th. Opened another abscess over the ribs. He progressed slowly from this time up to the 14th of June, when he was able to leave for the seaside. The abscess still discharging. The change much improved his general health, but the abscess did not completely heal until the end of October.

In the *Guy's Hospital Reports* for 1874, Vol. 19, several cases are recorded by the late Dr. C. Hilton Fagge, but no mention is made of this kind of abscess in any of the text-books I consulted; and even the *Medical Digest* does not refer to the series of cases in the *Guy's Hospital Reports*.

## Figures and Tables

**Figure f1:**